# Emergence of HGF/SF-Induced Coordinated Cellular Motility

**DOI:** 10.1371/journal.pone.0044671

**Published:** 2012-09-06

**Authors:** Assaf Zaritsky, Sari Natan, Eshel Ben-Jacob, Ilan Tsarfaty

**Affiliations:** 1 Blavatnik School of Computer Science, Tel Aviv University, Tel Aviv, Israel; 2 Department of Clinical Microbiology and Immunology, Sackler School of Medicine, Tel Aviv University, Tel Aviv, Israel; 3 School of Physics and Astronomy, The Raymond and Beverly Sackler Faculty of Exact Sciences, Tel-Aviv University, Tel-Aviv, Israel; 4 Center for Theoretical Biological Physics, Rice University, Houston, Texas, United States of America; 5 Research & Development Unit Assaf Harofeh Medical Center, Zerifin, Israel; 6 School of Physics and Astronomy, Tel Aviv University, Tel Aviv, Israel; University of Edinburgh, United Kingdom

## Abstract

Collective cell migration plays a major role in embryonic morphogenesis, tissue remodeling, wound repair and cancer invasion. Despite many decades of extensive investigations, only few analytical tools have been developed to enhance the biological understanding of this important phenomenon. Here we present a novel quantitative approach to analyze long term kinetics of bright field time-lapse wound healing. Fully-automated spatiotemporal measures and visualization of cells' motility and implicit morphology were proven to be sound, repetitive and highly informative compared to single-cell tracking analysis. We study cellular collective migration induced by tyrosine kinase-growth factor signaling (Met-Hepatocyte Growth Factor/Scatter Factor (HGF/SF)). Our quantitative approach is applied to demonstrate that collective migration of the adenocarcinoma cell lines is characterized by simple morpho-kinetics. HGF/SF induces complex morpho-kinetic coordinated collective migration: cells at the front move faster and are more spread than those further away from the wound edge. As the wound heals, distant cells gradually accelerate and enhance spread and elongation –resembling the epithelial to mesenchymal transition (EMT), and then the cells become more spread and maintain higher velocity than cells located closer to the wound. Finally, upon wound closure, front cells halt, shrink and round up (resembling mesenchymal to epithelial transition (MET) phenotype) while distant cells undergo the same process gradually. Met inhibition experiments further validate that Met signaling dramatically alters the morpho-kinetic dynamics of the healing wound. Machine-learning classification was applied to demonstrate the generalization of our findings, revealing even subtle changes in motility patterns induced by Met-inhibition. It is concluded that activation of Met-signaling induces an elaborated model in which cells lead a coordinated increased motility along with gradual differentiation-based collective cell motility dynamics. Our quantitative phenotypes may guide future investigation on the molecular and cellular mechanisms of tyrosine kinase-induced coordinate cell motility and morphogenesis in metastasis.

## Introduction

Collective cell migration is prevalent in many physiological phenomena and is the most common motility pattern in living organisms [1]. In morphogenesis, large clusters of cells travel long distances to reach their ultimate biological destination. In tissue repair, sheets of cells move coordinately to repair damaged tissue. In cancer, cells invade the extracellular matrix and traverse across normal tissue with extreme efficiency to form metastases.

Extensive research has been carried out for many years in various experimental model systems to investigate, describe, analyze, model and simulate collective cell migration. There are several theories concerning the mechanisms behind collective motility [2], [3]. A relatively common one regarding the physical interactions is "Follow the Leader" [4], were cells at the leading edge are assumed to produce force to pull passive followers from cells located further away from the front [5], [6], [7], [8], [9]. For example, Inaki *et al.* [9] recently demonstrated that directionality can be encoded within a group of cells by the constituents attaining different signaling levels.

However, accumulating evidence implies that the behavior is more complex. Modern microscopy [10] revealed that distant cells extend in what is referred to as ‘cryptic’ lamellipodia against the substratum beneath their preceding cells, evidence that the cells further behind the leading edge do not simply act as naïve followers. Recent measurements of distributions of traction- and intercellular-forces within the monolayer also suggest that the "follow the leader" paradigm is too simplistic [11], [12], [13], [14] and argue that cells further away from the front are also self-propelled in the collective motility process. Several mathematical models have been devised to describe collective migration based on single cell motility and cell-cell interactions [15], [16], [17].

Another theory assumes that cell proliferation expands the colony and thereby generates pressures that cause the leading cells to move [5], [18], [19], [20], but earlier studies demonstrated that cell migration in mucosal healing is largely independent of proliferation [20]. Moreover, Poujade *et al*. [5] showed that proliferation can occur almost exclusively in the void regions and hence cannot provide complete explanation for the general phenomenon.

In the standard *in-vitro* wound healing assay, collective migration of cells toward the wound is induced by a sudden injury created by removal of a sheet of cells from the monolayer [21]. Traditionally, the assay is applied to measure the change in healing rate caused by chemicals, other environmental conditions or cell types.

The epithelial to mesenchymal transition (EMT) activated by alternations in gene expression regulates epithelial plasticity during morphogenesis, tissue repair and cancer invasion [22]. During EMT, epithelial cells become motile and invasive, a process that is characterized by an elongated and more spread morphology throughout [23]. Cancer metastasis consists of a sequential series of events, and the EMT and mesenchymal-epithelial transition (MET) are recognized as critical events for metastasis of carcinomas [24]. A current area of focus is the histopathological similarity between primary and metastatic tumors, and MET at sites of metastases has been postulated as part of the process of metastatic tumor formation [24].

Understanding collective cell motility and how it may lead to metastatic formation is an important task since the vast majority of cancer deaths result of progression from a localized lesion to distant metastases [25]. *In vitro* collective migration is common in breast cancer, as well as in many other cancer types [26]. Several signal transduction pathways and proteins that are related to collective processes in morphogenesis contribute to cancer progression, but their molecular action mechanisms remain mostly unknown [1]. Many efforts are invested in targeting the tyrosine kinase growth factor receptor Met and its ligand HGF/SF, the master regulators of cell motility in normal and malignant processes [27], [28], [29], [30].

Here, we investigate the link between cells' morpho-kinetic dynamics and collective migration of tumor cells using mammary adenocarcinoma cells expressing high levels of Met, image them using a time-lapse microscopy wound healing assay, and study the effect of HGF/SF-Met signaling on morphology and collective motility patterns.

We devised a novel approach to analyze wound healing *in vitro* using bright field, time-lapse microscopy, based on the combination of a fully automated algorithm that extracts motility measurements from all cells in the monolayer with indirect cellular morphology measures using image-texture descriptors, and single cells' morphology measurements extracted semi-manually. Recording these as a function of location over prolonged periods allows a coherent and concise depiction of the essence behind three collective motility modes of cancerous cells, and to reveal that Met-activation by HGF/SF induces elaborated collective cell motility that is correlative to the EMT-MET morphological transition.

## Materials and Methods

### Cell Lines/Growth

DA3 cells, derived from the mouse mammary adenocarcinoma cell line D1-DMBA-3, induced in BALB/C mice by dimethylbenzanthracene [31] were grown in DMEM supplemented with 10% heat-inactivated FCS (Gibco ± BRL).

### Wound Healing Assay

DA3 cells expressing the fluorescent protein mCherry were grown to 90% confluence in wells of 2 cm^2^ diameter and treated with or without the Met inhibitor PHA665752 [32] (2.5 µM) for 2 hours. A scratch of approximately 300 µm in width was generated using a 200 µl tip, and the cells were incubated with or without HGF/SF (80 ng ml^−1^) and subjected to time lapse confocal laser scanning microscopy (CLSM-510, Zeiss, Germany) for approximately 26 hours, at frequency of once every 14.5 minutes. The position of each scratch was predefined, and a macro that repetitively positions the microscope on each point was executed. The acquired differential interference contrast (DIC) channel of the time-lapse sequence was used for the multi-cellular analysis; the red fluorescence channel was exploited for single cell tracking.

#### Phases in the healing process

**Figure 1:**
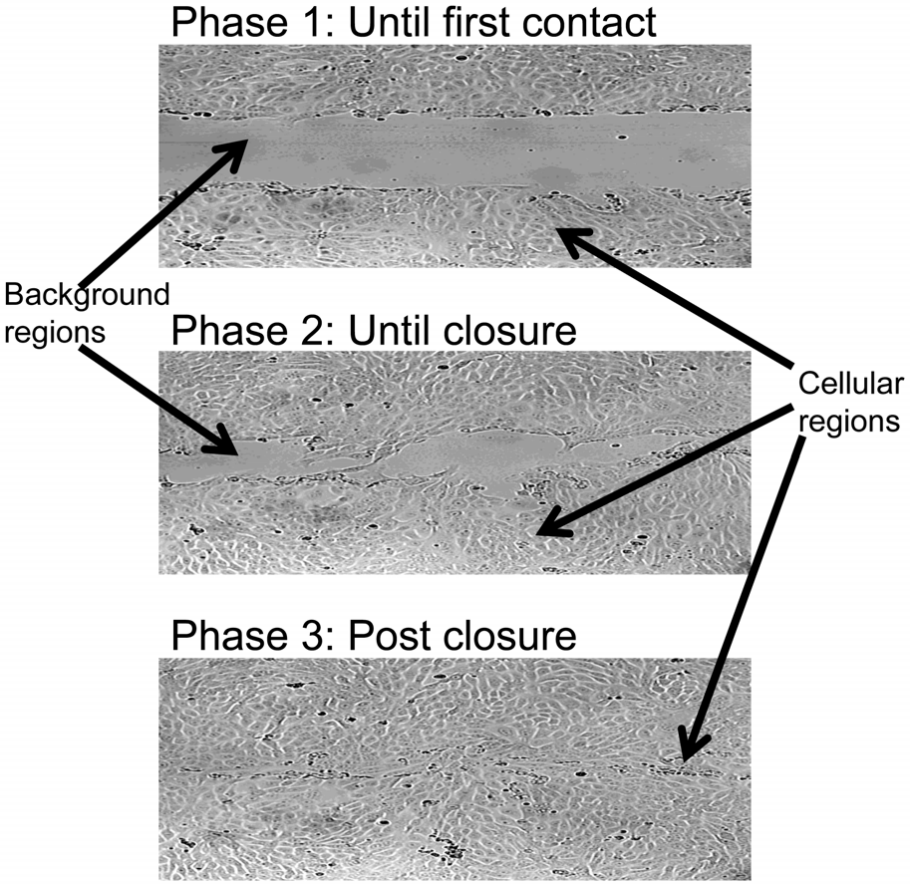
Three phases in the healing process. *Phase 1*: From the first frame in the time-lapse sequence, until first contact between cells from opposing edges of the wound. *Phase 2*: until full closure of the wound. *Phase 3*: post wound closure.

Three phases were defined (Figure 1). *Phase 1*, from the scratch formation until first contact between cells from opposing wound edges; *Phase 2*, from first contact until full closure of the wound - the wound area is completely covered by one layer of cells; *Phase 3*, post wound closure. The effect of HGF/SF on cellular motility patterns, morphology and multi-cellular texture were evaluated according to the three healing phases. Dividing the healing process to phases is important since the duration of each phase varies between the treatments.

### Velocity Magnitude Map – Fully Automated, Objective, Multi-Cellular Motility Visualization and Representation

Motility measurements were extracted via a fully-automated algorithm that quantifies local motion estimation from the time-lapse bright field (DIC) channel. The algorithm includes segmentation to partition a DIC image to multi-cellular- and background- regions, followed by local-motion estimation and quantification of the extracted motion fields' magnitude (which resembles cells' local speed) at different distances from the wound edge. Continuous quantitative description of cells' velocity magnitude as a function of distance from the wound throughout the healing process is achieved by constructing a “*velocity magnitude map*”:

Given two consecutive DIC frames *t*, *t*+1 from the time-lapse sequence.

**Figure 2:**
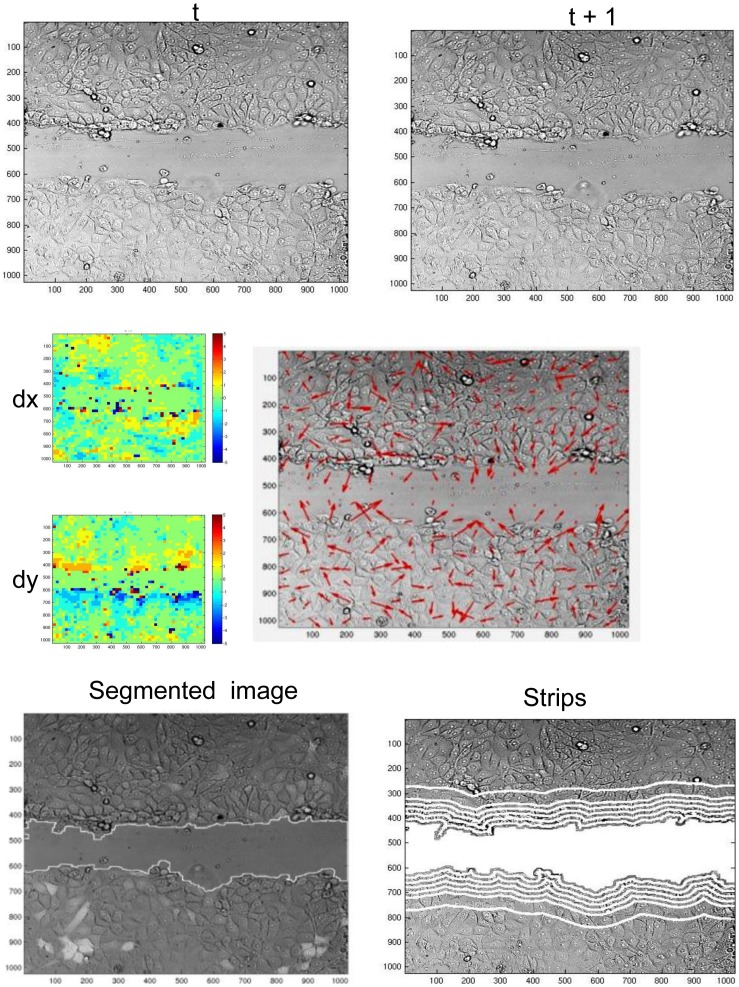
Motion estimation. Given two consecutive frames from the DIC time-lapse sequence (upper row), local-motion estimation is performed at the patch level (of size ∼18.5 µm×18.5 µm each) to produce the velocity-estimation vector fields. In the right part of the middle row, dx, dy are the partition to the two motion-components (dy – toward the wound, dx – parallel to the wound), the velocity fields are explicitly represented in the large image in the middle row. The segmented image (lower-left corner) displays the contours extracted by the MultiCellSeg algorithm over the DIC image. The wounded regions are used to define *strip*, a mask containing all pixels in a given distance from the wound (bottom-right). To quantify the "average" cell's motility at a given distance from the wound, velocity magnitude is averaged over the corresponding mask.

- Partition the current image (at time *t*) to a grid of sub-cellular sized local patches.
- Apply motion estimation to retrieve velocity fields estimations for each patch (Figure 2, top rows).
- Segment the image to cellular and background regions, and use the segmented image to define *strips*, mask containing all pixels at a given distance from the wound edge (Figure 2, bottom rows).
- For a given distance *d* from the wound edge, calculate the speed of the "average" cell located at *d* by averaging the velocity magnitude of all pixels in the corresponding strip. This step is repeated for every *d*.

**Figure 3:**
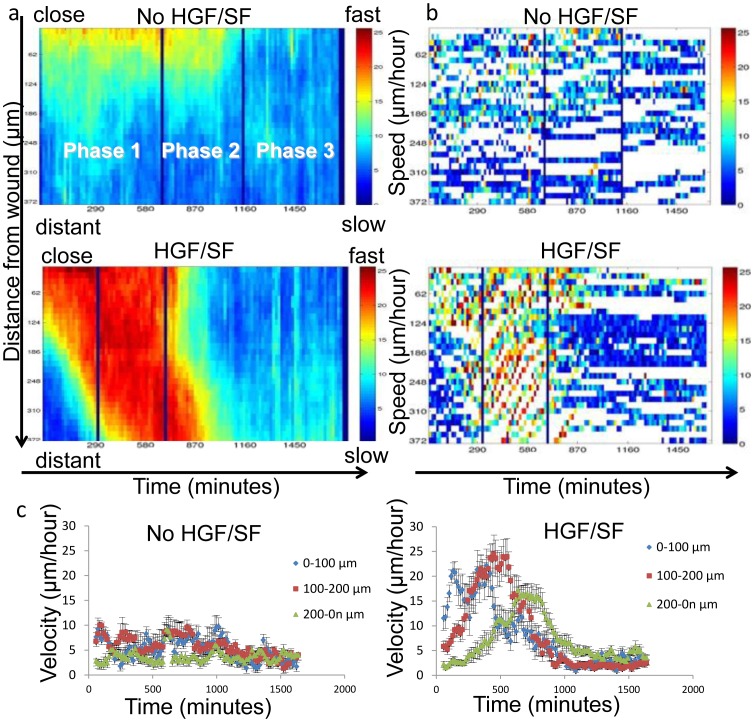
Velocity Magnitude Maps. (a) A two-dimensional depiction of the average motility of all cells at a given distance from the wound edge (*y*-axis) at a given time (*x*-axis). Each bin (*t*,*d*) represents the average motility (µm hour^−1^) of all cells at distance *d* from the wound at time *t*. Examples of two representative velocity magnitude maps are shown: untreated and HGF/SF-treated. The two vertical lines in each map define the partition to the three phases in the healing process. (b) The maps constructed from single-cell tracking. Examples of untreated and HGF/SF-treated cells are displayed. Comparison with the corresponding multi-cellular maps reveals that this approach provides a significant advantage over single-cell analysis. (c) Single cell tracking at several distances from the wound. Only the velocity component toward the wound is considered.

Examples of two representative velocity magnitude maps, of HGF/SF-treated and untreated cells are presented in Figure 3a; the two vertical lines define the partition to the three healing phases. Detailed description of each step in velocity magnitude map's construction is found in the Methods S1.

### Multi-Cellular Texture Representation

Similarly to the velocity map, each image is represented by the histogram of texture-descriptors in its cellular regions (defined by the segmented image). The Local-Binary Patterns (LBP), known to perform well in face-recognition [33], were applied as the texture descriptor. It is a gray-scale invariant texture measure: for every pixel, a code is generated based on the number and location of neighboring pixels with higher and lower intensities than that pixel. There are ten possible codes, and their histogram over all cellular pixels is used to describe the image's texture (Figure S1), an implicit measure for cells’ morphology that is similar to the one for cell scattering [34]. Thus, a time-lapse experiment is again represented by a two-dimensional map: the x-axis represents time, whereas the y-axis is the LBP histogram.

### Treatment Prediction for Wound Healing Time-Lapse Experiments

To enable automatic prediction whether a full DIC time lapse wound healing experiment was or was not treated with HGF/SF, the 3-dimensional time-lapse (image space and time) was "compressed" to a 2-dimensional representation, the velocity magnitude map described above. This compact description was further represented by a one-dimensional descriptor vector as follows: distances from the wound were partitioned to 6 intervals, and the average speed of all cells in any given interval during the 3 phases in the healing process were used to define a vector representation of a time lapse wound healing experiment (Figure S2a). To cancel out the effect of the general increased motility induced by HGF/SF, a second representation was achieved similarly, by normalizing these vectors to be of norm 1. Similar predictions were performed with multi-cellular texture representation (Figure S2b), using the average texture-descriptor (described above) from first contact between cells from opposing edges of the wound until full closure is achieved (*Phase 2*).

## Results

### Motility Analysis

Qualitative comparison of the velocity magnitude maps visualization between untreated and treated cells revealed the unique motility patterns induced by HGF/SF (Figure 3a). Front layers of untreated DA3 cells move faster than those located behind, demonstrating a homogeneous motility pattern during the wound healing process (*Phases 1* and *2*). During post wound closure (*Phase 3*), all cells decelerate regardless of their position. HGF/SF treatment leads to emergence of dramatic different cell motility patterns: at the beginning, front cells move faster than distant cells. Throughout *Phase 1*, distant cells gradually join the rapid motion by accelerating layer by layer. This gradual acceleration continues during *Phase 2*, where distant cells maintain a significantly higher motility toward the wound than cells located closer to the wound edge (data not shown). Finally, post wound closure (*Phase 3*), front cells halt, while distant cells gradually decelerate. These results demonstrate that Met-activation via HGF/SF induces complex motility patterns indicating cell-cell coordination and dynamic signaling that generates micro-differentiation in the healing wound.

**Figure 4:**
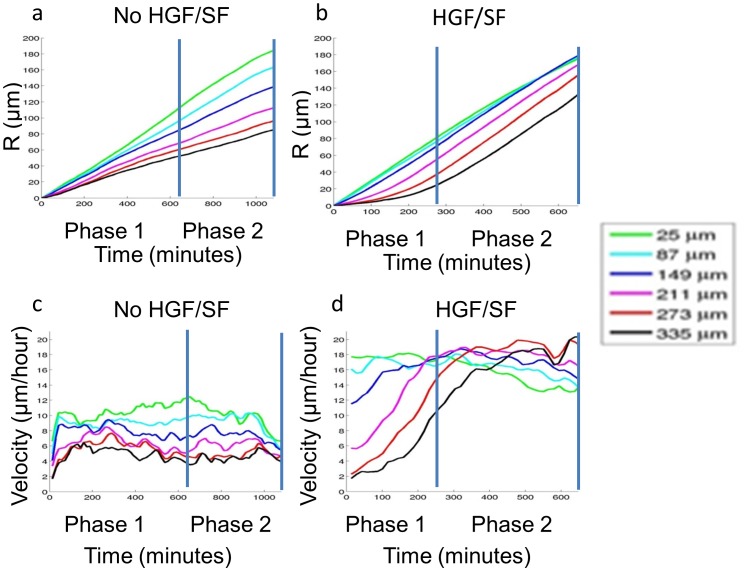
Motility patterns. (a–b) “Average” cell tracking toward the wound, and the displacement gap mystery. An “average” cell’s velocity at a given time and distance from the wound is defined as the average velocity component toward the wound in the strip that corresponds to the relevant location. The distance that an "average" cell travels in each frame (retrieved from the corresponding velocity fields) was accumulated to define its displacement as function of time. The *x*-axis represents time; the *y*-axis represents the “average” cell’s displacement toward the wound at several spatial locations. For untreated cells (a) it is demonstrated that front cells accumulate an expanding displacement gap over distant cells during healing. For HGF/SF-treated “average” cell tracking (b). A gap is formed between front and distant cells; however, during *Phase 2* it is shown that cells from behind progressively accelerate so that the displacement-gap formed at *Phase 1* shrinks for distant cells. (c–d) Untreated cells (c) exhibit roughly constant velocity toward the wound, whereas close cells are faster than farther cells. Distant HGF/SF-treated cells (d) exhibit gradual acceleration until they maintain higher velocity toward the wound than close cells in *Phase 2*.

To further demonstrate these phenomena, an alternative visualization is presented in Figure 4a–b, taking into account the direction perpendicular to (toward) the scratch. "Average" cells (calculated as indicated in Materials & Methods) at several locations (25–335 µm from the wound edge) were selected and "tracked" throughout the healing process. The distance that an "average" cell travels in each frame (retrieved from the velocity fields' estimation) was accumulated to define its displacement as function of time. Indeed, this measure is not exactly the actual displacement, as it contains algorithmic "noise", cells deformations and proliferation. Nevertheless, since the errors and noise occur in all "directions", we assume that this measure is an approximated representation of the true dynamics. Figure 4a–b displays this displacement measure (denoted R) as a function of time until full closure (*Phases 1* and *2*). It is shown (Figure 4a) that untreated cells express a "fan-like" dynamics, where front cells expand a physical gap from cells behind, a gap that grows steadily during healing. As for treated cells (Figure 4b): during *Phase 1*, a gap is formed between front and distant cells, but cells from behind progressively accelerate so that this displacement-gap shrinks or at least remains constant during *Phase 2* for cells located about 10 cell-layers behind the leading edge. Figure 4c–d plots the average velocity component toward the wound over time. Untreated cells exhibit roughly constant velocity, front cells being faster than farther cells (Figure 4c). The gradual acceleration of distant HGF/SF-treated cells throughout *Phase 1*, and the higher velocity maintained by distant cells compared to front cells is displayed in Figure 4d. These conclusions referring to an estimation of the "average" cells' velocity over time are supported by single-cell tracking experiments as shown in Figure 3c.

**Figure 5:**
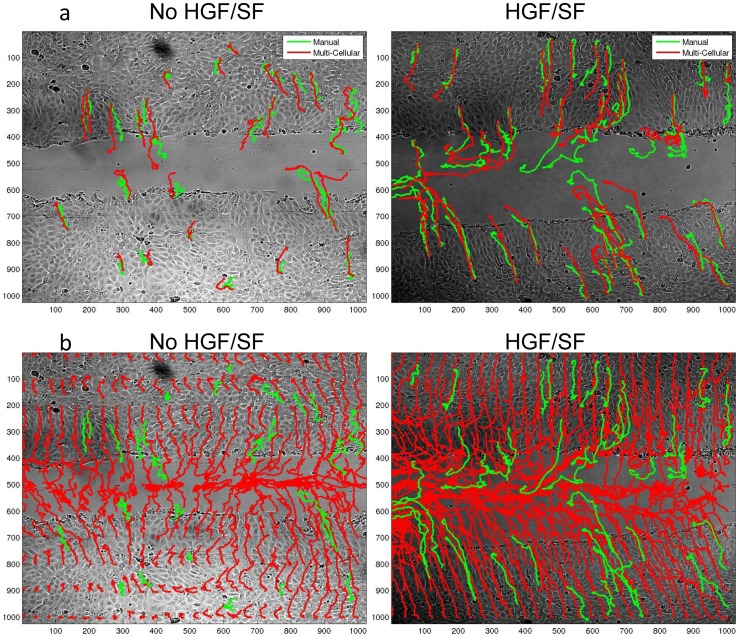
Multi-cellular DIC based single cell trajectory estimation. (a) Visual comparison of manually tracked cells (green) and automated trajectories extracted from local DIC-based motion estimation (red). It is shown that the automated trajectories are highly correlated to the manually-validated trajectories. Examples of untreated (left pane) and HGF/SF-treated (right pane) are illustrated. (b) Visual illustration of the advantage in using all cells' information in comparison to part of the cells. Green trajectories are the manually-validated trajectories, red are trajectories extracted by our method. Left pane - untreated cells, right pane - treated cells.

To validate the local motion-estimation, which is a fundamental component in our analysis, we compared manually-validated fluorescence-based semi-automated single cells tracking to fully-automated single-cell trajectory estimation extracted using these local motion-fields (as described in Methods S1). These trajectories are highly correlative to the manually-validated trajectories (Figure 5a). Moreover, examinations of corresponding multi-cellular versus single-cell based velocity maps (Figure 3a-b) clearly demonstrate a significant qualitative advantage of the former approach: using noisy estimation of **all** cells (Figure 5b) enables an enhanced and a more coherent representation of the true nature of the process.

### Single-Cell Morphology Analysis

**Figure 6:**
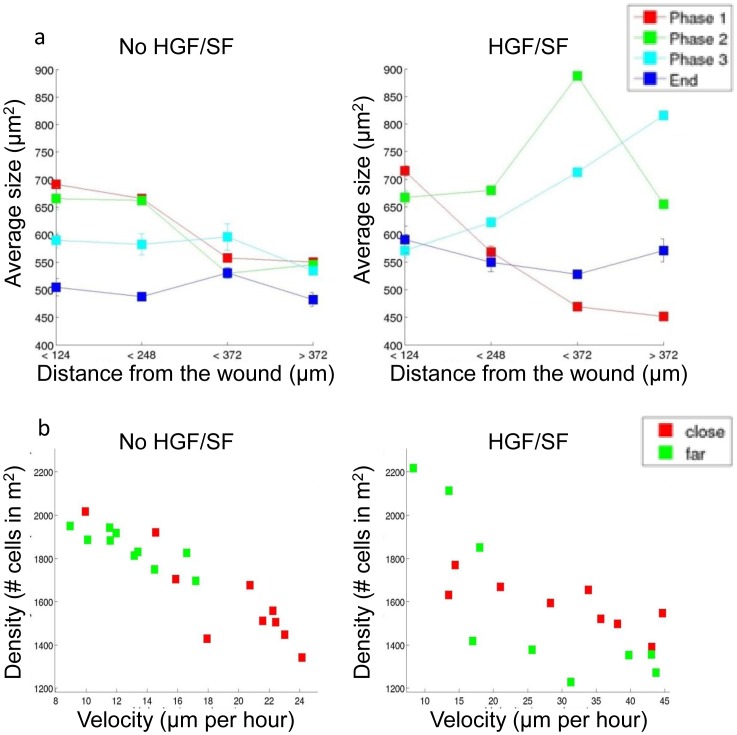
Single cell morphology (area) as function of time and distance from the wound. (a) “Average” cell’s area at different distances over time. Untreated (left), and HGF/SF-treated (right) cells. The x-coordinates represent discrete distance-intervals from the wound edge, the y-coordinates are the average cells’ size at a given distance interval and at a given phase in the healing process. Color markers represent the phase in the healing process: from the initial scratch until first contact between cells from opposing borders of the wound (*Phase 1*, red), until full closure (*Phase 2*, green), post wound closure (*Phase 3*, pale blue), and last frame in the time lapse sequence (∼26 hours after the initial scratch, dark blue). The analysis demonstrates that HGF/SF induces dramatic morphological changes at the single cell level. (b) Relation between cells density and speed for untreated (left) and treated (right) cells. The cells’ density is estimated at two spatial location <248 µm (marked red), and >248 µm (marked green) from the wound edge. Based on the single cell’s area statistical analysis, speed was calculated in the same distance intervals from the velocity magnitude map. Correlation significance between velocity and density was calculated with the non-parametric Spearman's rank correlation coefficient. This analysis demonstrates a significant correlation between density and velocity with no dependency on the spatial location. This correlation is less prominent for treated cells (p<0.003, compared to p<0.0001 for untreated cells) although still statistically significant.

Figure 6a illustrates the average cell's area as function of distance from the wound over the different healing phases for untreated (left) and treated (right) cells (as described in Methods S1). In addition to the predefined three healing phases, a fourth time point was added, which represents the last frame in the time-lapse and is used to demonstrate the final stages of the healing process. The x-axis consists of 4 different distance intervals from the wound edge, the y-axis is the average cells' area at a given phase and distance-interval. Until full closure (*Phases 1* and *2*) untreated cells that are close to the wound are larger than distant cells. Front cells shrink upon wound closure, and after the wound has healed all cells in the monolayer shrink to maintain approximately the same size independently of their location. Similarly to its effect on cells' speed, HGF/SF treatment induces dramatic changes in the dynamics of cellular morphology. At the initial stage, close cells are larger than distant cells. During *Phase 2*, front cells begin to shrink while farther cells grow. In *Phase 3*, only the most distant cells continue to grow while the rest shrink. After the wound has healed, all cells have shrunk to approximately the same size. A bar graph that compares treated and untreated cells' area for every distance interval over time demonstrates that the main differences occur in *Phases 2* and *3*, when treated cells that are located far from the leading edge grow dramatically in a progressive manner (Figure S3a). Similar patterns of morphology alteration are depicted using cell eccentricity. Untreated cells that are close to the wound are more elliptical than distant cells throughout the healing, while front treated cells begin as more elliptical than distant cells, that in turn, during the later stages, become more elliptical than these front cells (Figure S4).

### Cells Density and Motility

Next, the relation between cell motility and density was examined. Cells density was estimated based on cell size measurements as detailed in Methods S1. Throughout the healing process, untreated cells that are close to the wound’s edge (<248 µm) are consistently spread sparsely compared to distance cells (>248 µm) (Figure S3b, left). Treated cells maintain similar location-dependent characteristics to those described for untreated cells during *Phase 1*. However, upon *Phase 2*, treated cells "switch" - distant cells become sparsely distributed compared to front cells (Figure S3b, right). Investigating cells’ density and motility reveals that, as expected, sparser regions are highly correlated with faster velocities, as was recently shown [8], [12], independently of cells’ location, and is more prominent for untreated cells (Figure 6b).

### Generalization: Prediction of Treatment Based on Motion and Texture Patterns

**Figure 7:**
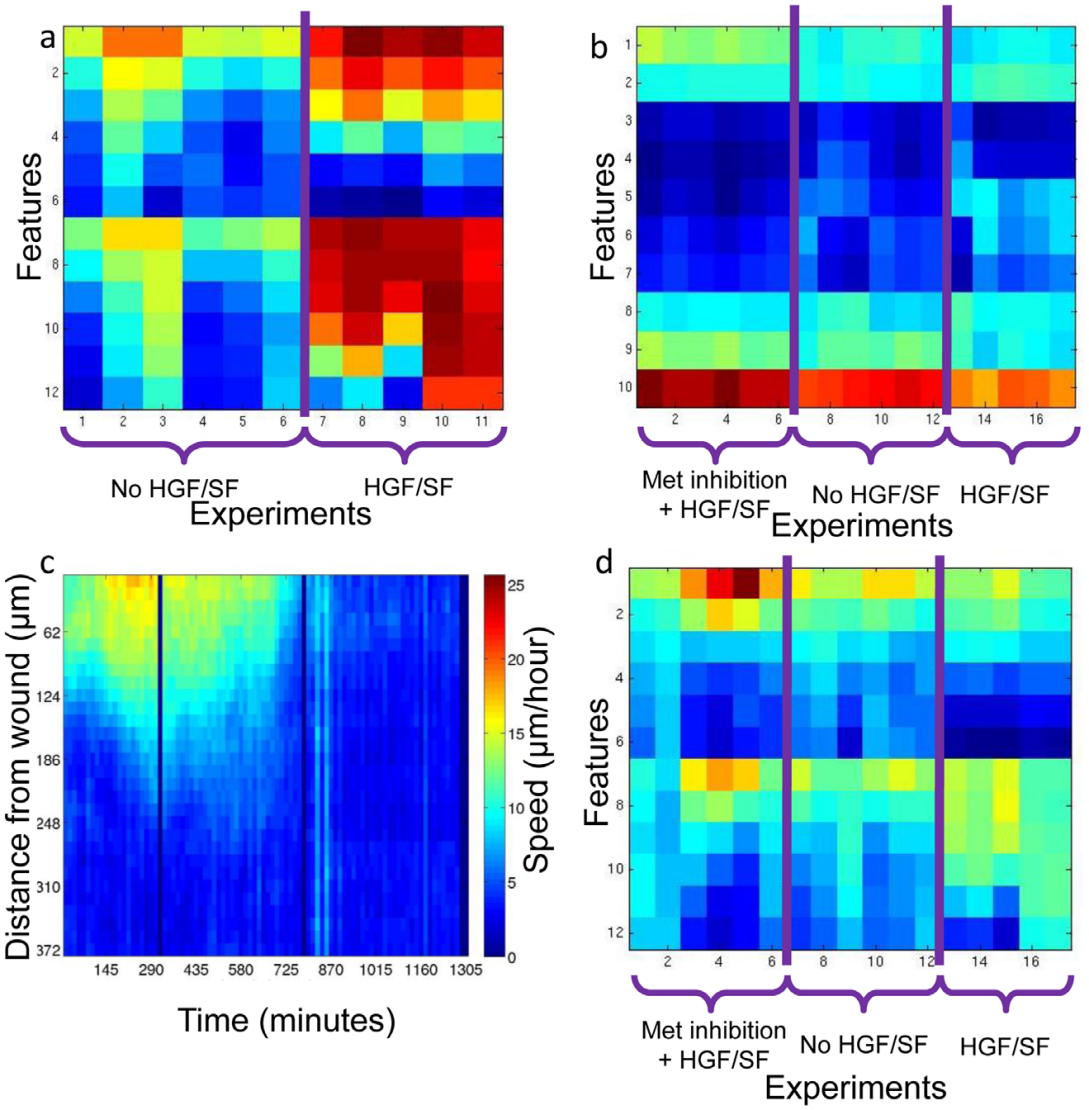
Generalization: multi-cellular speed- and texture-based classification. (a) Velocity magnitude-based vector-representation of a full time lapse sequence. Each column represents a single experiment. The vector values were calculated as the average velocity magnitude of all cells at a given distance-interval from the wound, at a given phase. The analysis demonstrates that the first six experiments (untreated) are very different from the last five (HGF/SF-treated). (b) Texture-based vector-representation of a full time lapse sequence. The LBP image-texture descriptor normalized histogram is averaged over all time frames from *Phase 2*, when most morphological changes occur. Each column is the LBP histogram extracted from a single experiment. It was demonstrated that there exists a clear discrimination between any pair of the three conditions: untreated, HGF/SF, and Met inhibition+ HGF/SF. (c) Example of a velocity magnitude map of cells treated with Met inhibition and HGF/SF. (d) Collective motility patterns of full time lapse experiments. Each column represents the normalized spatio-temporal velocity magnitude of a single experiment. It was demonstrated that there exists a clear discrimination in collective motility patterns between any pair of the three conditions: untreated, HGF/SF, and Met inhibition + HGF/SF, as Met-signaling becomes more active, the ratio between motility of distant cells and close cells decreases which implies that cells located farther from the wound become more active by Met-signaling activation.

To generalize these findings, we tested whether cells in wound healing assays can be automatically classified as HGF/SF-treated or -untreated based solely on the DIC time-lapse images. To this end, we defined a new measure to quantify the collective motility patterns: each of the 11 time lapse experiments (6 untreated, 5 treated with HGF/SF) was represented by a vector containing average speed for each healing phase as described in the Materials and Methods section (Figure S2a). An SVM classifier was trained and tested using "leave one out" validation (due to the small number of experiments obtained), and a 100% accuracy rate was achieved. Hence, an accurate prediction can be reached based on the DIC time lapse velocity-estimation alone. It is not surprising, since there is a clear visual separation between the motility patterns for treated and untreated cells (Figure 7a). To examine the motility **patterns**, these vectors were normalized to rule out the general increase in velocity magnitude due to HGF/SF, a perfect prediction was achieved when considering *Phases 1* and *2* in the healing process (until full closure), implying that there is a true-general change in the motility patterns and not only in its magnitude. This means that given a time lapse experiment and two time points that represent the partitioning to the three phases, one can determine with high accuracy whether it was treated with HGF/SF or not (p<0.0043, using the a-parametric test Wilcoxon rank sum). Similar predictions were performed with multi-cellular texture descriptor (as described in the Materials and Methods section). It was shown (Figure 7b) that perfect classification is achieved by considering cellular texture at *Phase 2* (from first contact to full healing, where most morphological changes occur), applying an SVM classifier using "leave one out" validation (p<0.0043, the same a-parametric test).

### Effect of Met Inhibition on Velocity Magnitude Patterns

To investigate the molecular mechanism underlying collective motility and to examine the robustness of our measures, we examine the effect of Met-inhibition on the quantitative measure described above. Velocity magnitude maps of cells treated with the Met inhibitor and HGF/SF were extracted (e.g., Figure 7c), and two SVM-classifiers was trained and tested using "leave-one-out" validation to separate between (6 repeats of) cells treated with the Met inhibitor + HGF/SF and (1) untreated or (2) HGF/SF-treated cells. Since some of the experiments treated with the Met inhibitor + HGF/SF did not achieve full-closure (*Phase 2* was not completed), only the first two healing phases were considered (descriptor vector of length 12 per experiment). To exclude the global healing speed and to focus on the spatio-temporal motility patterns, each experiment descriptor was normalized to 1 (Figure 7d). Two-components PCA analysis was unable to discriminate between the treatments (Figure S2c). Perfect classification was achieved with each of the two classifiers to conclude that the motility patterns of cells treated with Met inhibitor and HGF/SF differ inherently than untreated (p<0.0022 using Wilcoxon rank sum a-parametric test) and HGF/SF-treated (p<0.0043 using the same test). As Met-signaling becomes more prominent, cells located farther than the wound take an active role in collective motility, Figure 7d shows that when treated with Met inhibition + HGF/SF the ratio between front cells' and distant cells motility is maximized, this ratio decreases for untreated cells and is minimal when Met-signaling is induced by HGF/SF. Perfect classification between all pairs of treatments was also demonstrated using the texture-representation (Figure 7b). These results indicate that the endogenously over expressed Met in these cells [27] plays a role in collective cell motility thus validating the involvement of the Met-signaling pathway with induction of collective motility patterns as well as the discriminative power of our proposed morpho-kinetic measurements.

## Discussion

**Figure 8:**
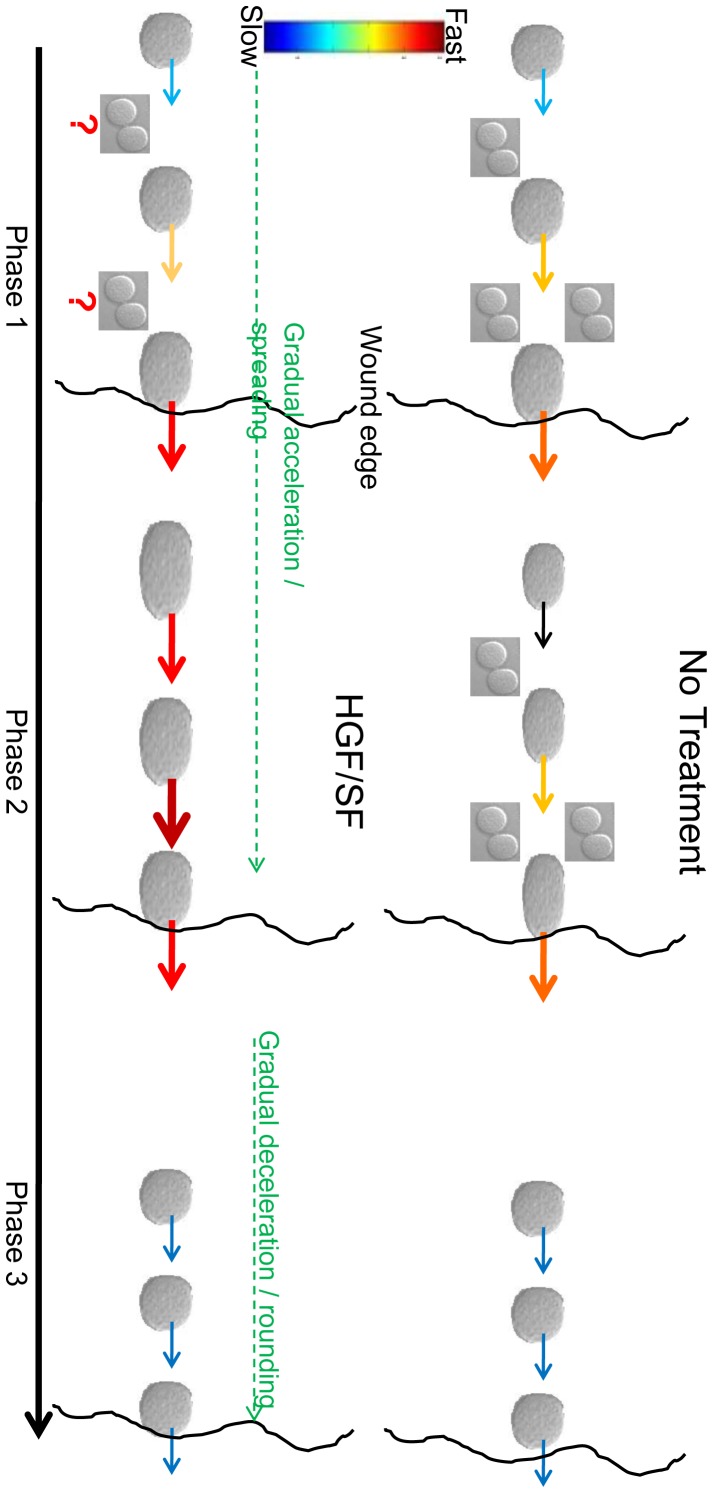
Model for HGF/SF-induced collective motility patterns during the healing process. HGF/SF dramatically alters the morpho-dynamics of the healing wound: from a simple model, in which the front cells lead the healing in constant acceleration, to a more elaborate model in which cells in different distances from the wound lead a coordinated increased motility along with spatio-temporal phenotypic EMT-MET based collective cell motility dynamics. Untreated DA3 front layers cells are larger, more elliptical and move faster (marked by wider arrows) than cells located behind demonstrating a homogeneous motility pattern during the wound healing process (*Phases 1* and *2*). During post wound closure (*Phase 3*), all cells decelerate, shrink and round up regardless of their position. HGF/SF treatment leads to the emergence of dramatic different cell motility patterns: at the beginning, front cells are larger, more elliptical and move faster than distant cells. Throughout *Phase 1*, distant cells become larger, more elliptical and gradually join the rapid motion by accelerating layer by layer. These morphology changes and gradual acceleration continues during *Phase 2*, were distant cells maintain a higher velocity toward the wound than cells located closer to the wound edge. Finally, post wound closure (*Phase 3*), front cells shrink, round up and halt, while distant cells gradually decelerate, and change morphology in a similar manner. It is hypothesized that accelerated proliferation at the leading edge is the answer for the untreated cells gap mystery presented in the text. It is hypothesized that in treated cells proliferation occurs more intensively, but is spread approximately equally throughout the monolayer.

Collective cell migration mechanisms are important for normal and pathological biological processes. We propose a quantitative hybrid measure that incorporates fully automated cellular spatiotemporal motility and indirect morphological measures together with semi-automated direct morphological measures to describe the kinetics of collective cells migration. Applying this analysis, we demonstrate that HGF/SF dramatically alters the morpho-kinetic dynamics of the healing wound: from a simple model in which the front cells lead the healing at constant speed; to a more elaborate model in which cells lead a coordinated increased motility along with spatiotemporal phenotypic EMT-MET-like collective cell motility dynamics (Figure 8). Met-inhibition experiments demonstrating inhibition of cell motility, validated the important role of HGF/SF-induced Met activation in breast cancer metastasis.

Traditionally, velocity fields are extracted by tracking individual cells during a time-lapse experiment [35]. Practically, single cell tracking in a monolayer requires considerable labor and can be usually performed only for a small number of cells, providing limited statistical coherency. Our method does not require single cell tracking nor fluorescent-based imaging and is fully-automated. The proposed collective cell migration morpho-kinetic analysis is based on local motion estimation, an approach well suited for DIC images, where inner cellular regions maintain high textural information enabling accurate motion-estimation at the patch level without further processing [8]. The main motivation behind it is the ability to process **all cells** within the monolayer; the dynamics of collective motility is complex, understanding the individual cell in more detail does not necessarily explain the collective kinetics of a monolayer of cells [36]. Another important advantage is the ability to be performed in high-throughput settings, such as suggested by Yarrow et al. [37].

Velocity magnitude maps are 2-dimensional continuous and compact representation of the local motion estimation vector fields during the entire time lapse wound healing experiment. Driscoll et al. [38] recently presented a similar visualization for the spatiotemporal evolution of a cell's boundary curvature. This concise and coherent visualization demonstrate the alteration of collective motility patterns induced by HGF/SF; we hypothesize that the treatment stimulates cells distant from the leading edge to become self-propelled in an organized and coordinated manner.

A major qualitative and quantitative utilization of velocity magnitude maps is the generalization of the change in collective motility patterns as a consequence of adding HGF/SF to the medium with or without Met inhibition. This phenomenon was validated qualitatively, by visual inspection of the velocity magnitude maps, and quantitatively by applying classification, treating them as plain images and extracting appropriate image-features. This measure allows perfect classification based solely on the motility patterns; the relative-role that cells take in collective migration as a function of their location. This means that given a full time lapse wound healing experiment, and two time points representing the three healing-phases, it is possible to predict with high accuracy the treatment applied to cells. This ability is a substantial improvement over the standard measures, usually only showing correlation between treatment and phenotype. Thus, we address Tambe et al.’s call [12], "…our understanding of collective cellular migration lacks predictive power and remains largely descriptive".

It is demonstrated that marking a small number of cells within a monolayer can be sufficient to reveal cellular morphology-dynamics. This analysis was applied to demonstrate the high correlation between cells’ morphology and motility: large and elongated cells are characterized by faster motility regardless of their spatial location or phase in the healing process. The dramatic coordinated morphological changes in HGF/SF-treated cells’ are another indication for being self-propelled. We hypothesize that the lower (although still significant) morphology-motility correlation of treated cells is explained by their self-propelled nature, causing lower dependence on their surroundings.

It was demonstrated here that texture of a single image from time lapse sequence, captured by the LBP descriptor, is sufficient to predict the treatment. The image-texture of a monolayer of cells can thus be used to implicitly measure cells morphological characteristics, as in measuring cell scattering [34]. We hypothesize that when considering large numbers of cells in a monolayer, relations between neighboring pixels’ intensities represent indirectly morphological characteristics of these cells. Indeed, the average texture descriptor of image frames from *Phase 2* discriminate between cells untreated, HGF/SF-treated and treated with MET inhibitor and HGF/SF together, concordant with the data indicating that the morphological changes mostly occur during this phase. Thus, using image-texture as an indirect multi-cellular morphology descriptor can be exploited as a treatment-predictor. Further investigation should try to find a direct connection between cells’ morphology and texture.

The combination of image texture and cellular velocity magnitude descriptors may serve as fully automated quantitative measures to represent morpho-kinetic dynamics, to enable in principle high throughput analyses without human intervention.

It is noteworthy that the *in vitro* model of tumor cells moving collectively studied herein does not take into account important parameters that maintain a crucial role in biological processes that include collective motility such as 3D motility and the tissue’s microenvironment (e.g., extracellular matrix resistance [39], [40], [41]). These parameters have prominent effects on collective cell migration in embryogenesis [1], tumor invasion [1], [42], and tubulogenesis [39], [40], [43]. However, important benefits in using *in vitro* models to study cellular and molecular mechanisms are controlling starting-point definition and ability to perform high-throughput screening and analysis [1].

The uneven velocity of DA3 cells in collective migration toward the wound *in vitro* is not associated with formation of finger-like structures [44] as in collective migration of MDCK cells [5]. The displacement gap formed between front and distant cells during untreated DA3 experiments, visualized by the fan-like dynamics (Figure 4a), contradicts the fact that the monolayer is kept continuous with no visible gaps throughout the healing process. Morphology transitions alone cannot account for this phenomenon, since cells' growth is insignificant in the gap formed by accumulating displacements between cells located closer and farther from the leading edge. On the other hand, the gap formed under HGF/SF treatment can be explained solely by morphology transitions; it is formed when cells near the wound’s edge become larger than distant cells. During the next phase, distant cells exceed the size of cells located closer to the leading edge and fill the gaps. We hypothesize that accelerated proliferation at the leading edge is the answer for the untreated cells’ “gap mystery”, as shown in Poujade *et al.* [5]. Since HGF/SF induces accelerated proliferation [45], we believe that it is spread approximately equally throughout the monolayer under treatment. This hypothesis, complementing the motility pattern description is also illustrated in Figure 8.

EMT is a process that changes proliferating cells from an aplanetic state to a motile state [46], [47], which allows cancer cells to leave the primary tumor and metastasize. The dramatic changes in cell morphology and behavior here is reminiscent of the EMT. It is thus likely that HGF/SF treatment leads to a more pronounced and accelerated morphological EMT, followed by an accelerated phenotypic MET post wound closure, which validates similar results regarding Met-induced EMT [48].

Analyses of Met-inhibited experiments indicate that endogenously-activated over-expressed Met plays an important role in collective cell motility and further validates involvement of Met-signaling pathway in this process. These results are coherent with recent findings by Loerke *et al* [49], connecting cell speed to cell-cell adhesion upon HGF/SF-induced Met-signaling.

Matsubayashi et al. have demonstrated that a "wave" of increasing velocities propagates back from the leading edge during monolayer wound healing of mIMCD3 mouse kidney epithelial cells [50]. Recently, Serra-Picamal *et al*. demonstrated a similar phenomenon for MDCK cells, also showing that stress forces gradually propagate from the leading edge backwards. They suggested that progressive cell mobilization away from the leading edge is a general response of cell collective motility [51]. We argue that this "wave" is induced by specific signal transduction; it is induced by HGF/SF, and reduced by Met-inhibition in breast cancer cells.

Collective motility should thus not be explained from a mechanical perspective alone; increased efforts should be invested in understanding the effects of various chemical signaling, which constitute a significant role in collective cell motility as demonstrated herein and in other studies (e.g., [9], [35]). Revealing the effect of Met-signaling on collective morpho-kinetic patterns is crucial to understand the molecular and cellular mechanisms behind metastasis. The ability to predict that a group of cells maintain a dynamical metastatic signature can have therapeutic implications in the long run; this may turn to be a first step in that exciting avenue.

## Supporting Information

Figure S1 **Multi-cellular texture-based classification.** Local Binary Patterns (LBP) applied as an image texture descriptor. For every pixel in the image, a code is generated based on the intensities of neighboring pixels with relation to it. There are ten possible codes, and their histogram over all cellular pixels is used to describe the image's texture which is used as an indirect descriptor of cells morphology.
(TIF)

Figure S2 **Compact representations of a wound healing experiment.** (a) Velocity-magnitude based vector representation of a wound healing experiment. Distances from the wound were partitioned to 6 intervals. The average motility of all cells in any given interval during each healing phase was recorded to define a length 18 vector representation. These values are calculated by taking the average intensities of the corresponding rectangular regions in the velocity magnitude map. (b) Texture-based vector representation of a wound healing experiment as an implicit measure for cells' morphology. LBP normalized histogram is extracted for every image in the time-lapse sequence. All histograms of frames in *Phase 2*, where most morphological-changes occur, are averaged to define the combined texture descriptor. (c) First two components of the principal component analysis (PCA) performed on the normalized velocity-magnitude based vector representation was not sufficient.
(TIF)

Figure S3 **Single cell morphology (area) as function of time and distance from the wound.** (a) “Average” cell’s area at different distances over time. Same data as presented in Figure 4, shown with different visualization. It can be seen that most morphological alterations occurs for HGF/SF-treated cells far from the wound at the later stages of healing. (b) Estimated density as function of time for close (<248 µm, red markers) and far (>248 µm, green markers) cells. Throughout the healing process, untreated cells that are close to the wound’s edges are consistently spread sparsely compared to distance cells. During *Phase 1* treated cells maintain similar location-dependent characteristics to those described for untreated cells. In *Phase 2*, treated cells "switch" - distant cells become sparsely distributed compared to front cells.
(TIF)

Figure S4 **Single cell morphology (eccentricity) as function of time and distance from the wound.** Eccentricity is the ratio of the distance between the foci of an ellipse and its major axis length. In our setting it is referred to the ellipse that has the same second-moments as the segmented cell. The values range between 0 and 1. (0 and 1 are degenerate cases; an ellipse whose eccentricity is 0 is actually a circle, while an ellipse whose eccentricity is 1 is a line segment.). (a) Same as Figure 6 a–b only for eccentricity instead of area: “Average” cell’s eccentricity at different distances over time. Untreated (left), and HGF/SF-treated cells (right). The x-coordinates represent discrete distance-intervals from the wound edge, the y-coordinates are the average cells’ eccentricity at a given distance interval and at a given phase in the healing process. Color markers represent the phase in the healing process. (b) Same as in Figure S3a only for eccentricity: “Average” cell’s eccentricity at different distances over time (different visualization).
(TIF)

Methods S1 **In this document we provide a detailed description of the following methods: (1) velocity magnitude map, (2) fully-automated single cell trajectory-estimation from DIC-based motion vector fields, (3) semi-automated single cell tracking, (4) velocity magnitude map based on semi-automated single cell tracking, and (5) single-cell morphology measures.**
(DOCX)
