# Supplementary figures and images for: Emergence of HGF/SF-Induced Coordinated Cellular Motility
Source: PLoS One. 2012 Sep 6;7(9):e44671. doi: 10.1371/journal.pone.0044671 (PMC3435317; doi:10.1371/journal.pone.0044671)

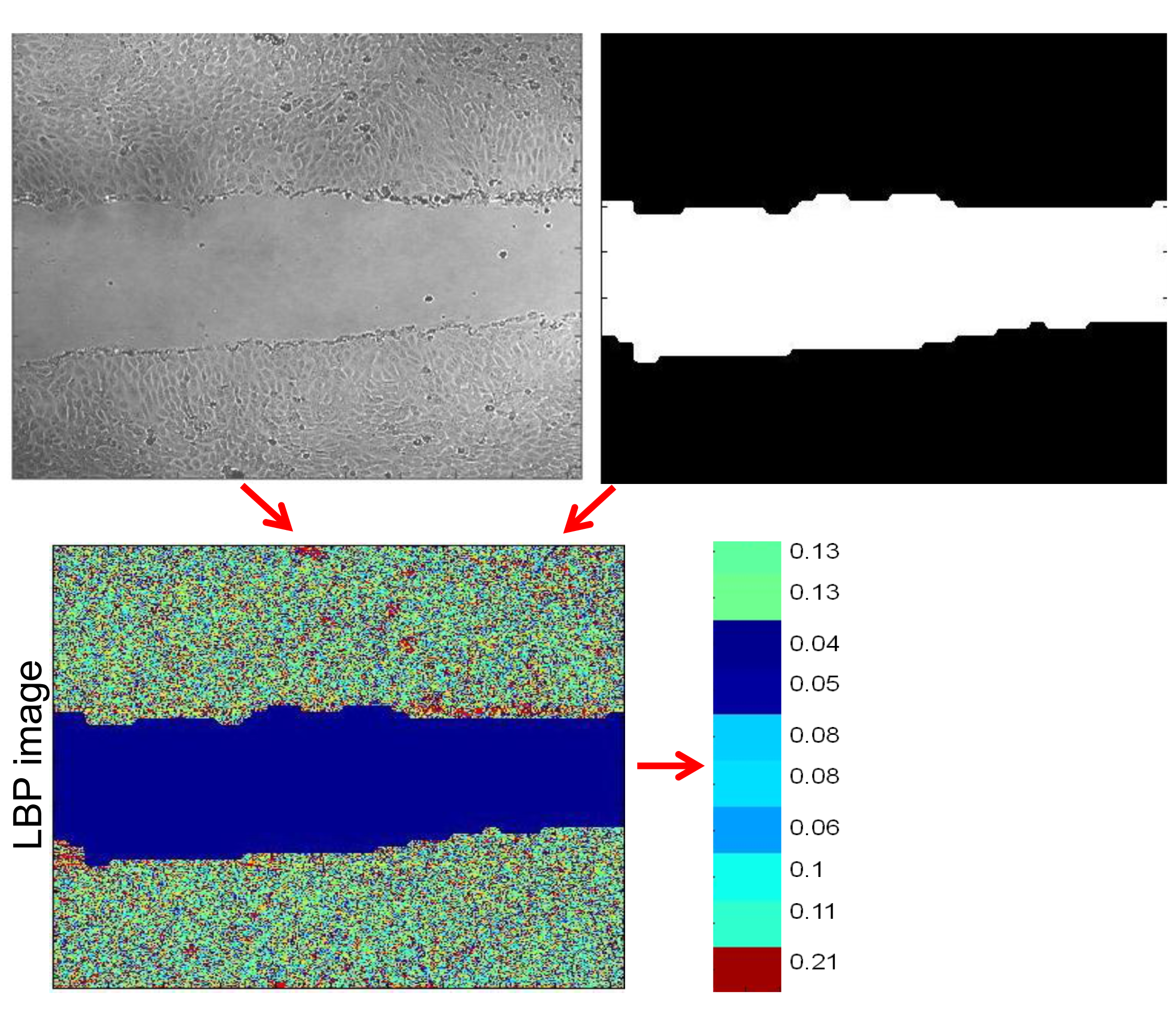

Supplement: Figure S1 — Multi-cellular texture-based classification. Local Binary Patterns (LBP) applied as an image texture descriptor. For every pixel in the image, a code is generated based on the intensities of neighboring pixels with relation to it. There are ten possible codes, and their histogram over all cellular pixels is used to describe the image's texture which is used as an indirect descriptor of cells morphology. (TIF) [file pone.0044671.s001.tif]

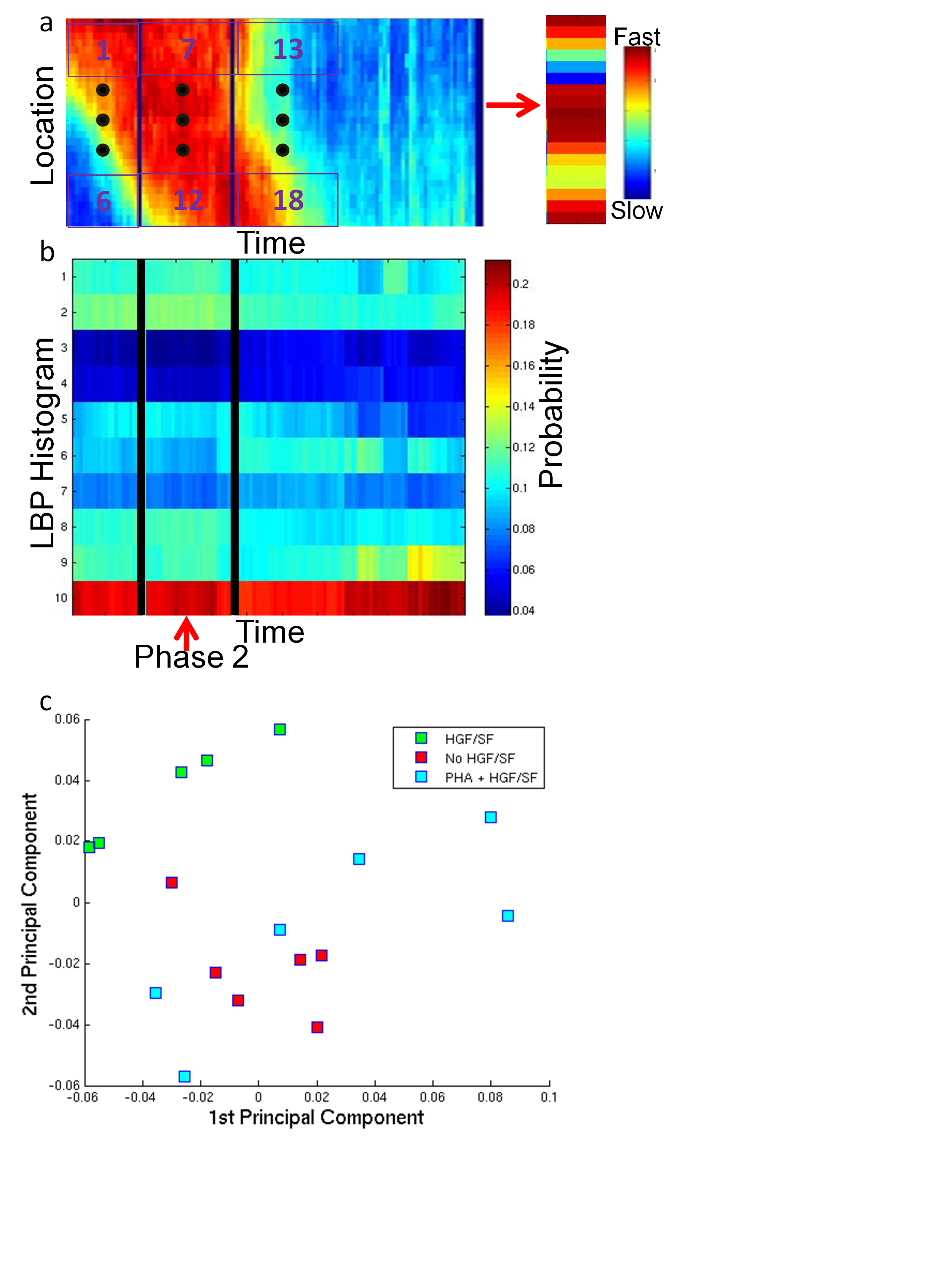

Supplement: Figure S2 — Compact representations of a wound healing experiment. (a) Velocity-magnitude based vector representation of a wound healing experiment. Distances from the wound were partitioned to 6 intervals. The average motility of all cells in any given interval during each healing phase was recorded to define a length 18 vector representation. These values are calculated by taking the average intensities of the corresponding rectangular regions in the velocity magnitude map. (b) Texture-based vector representation of a wound healing experiment as an implicit measure for cells' morphology. LBP normalized histogram is extracted for every image in the time-lapse sequence. All histograms of frames in Phase 2, where most morphological-changes occur, are averaged to define the combined texture descriptor. (c) First two components of the principal component analysis (PCA) performed on the normalized velocity-magnitude based vector representation was not sufficient. (TIF) [file pone.0044671.s002.tif]

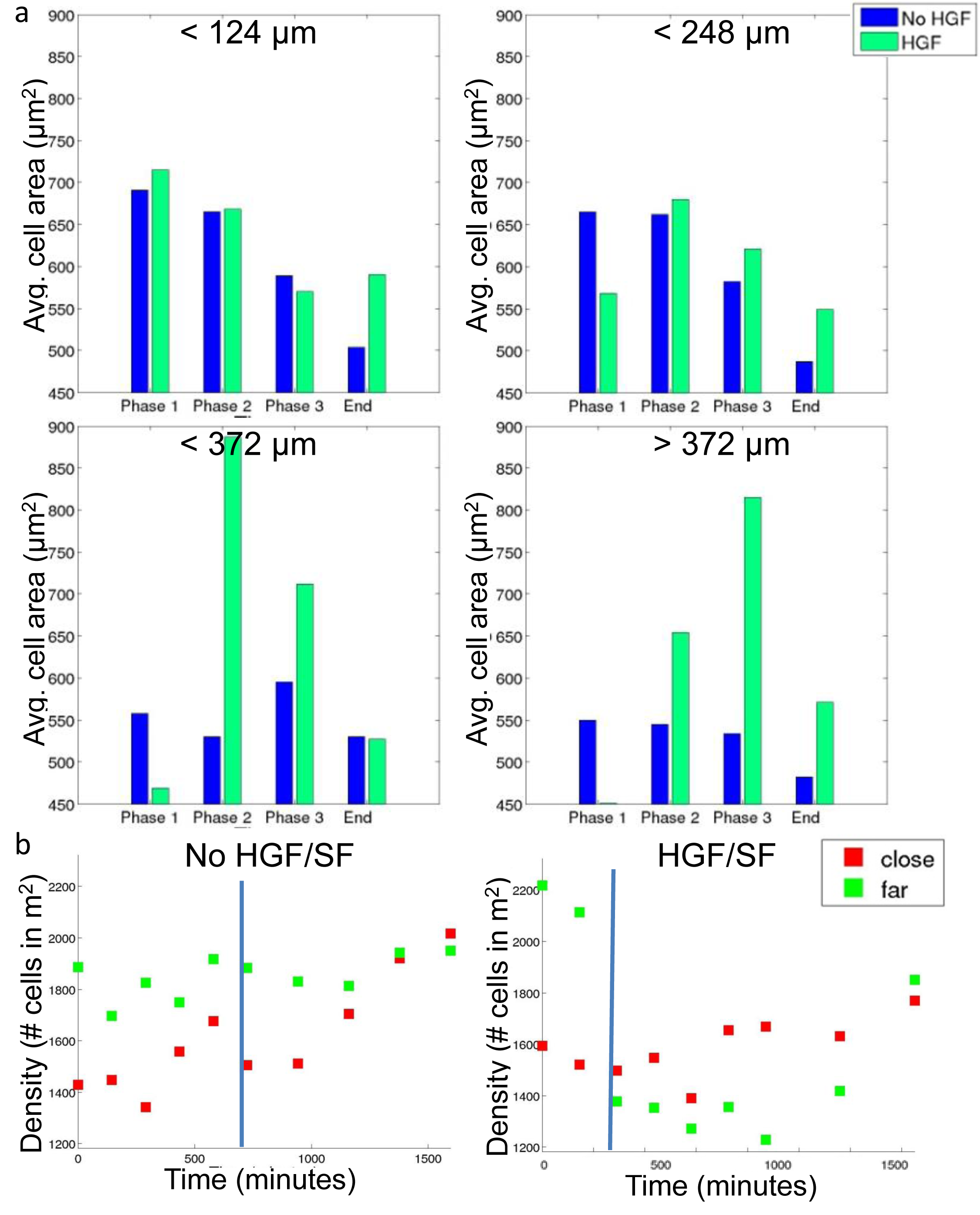

Supplement: Figure S3 — Single cell morphology (area) as function of time and distance from the wound. (a) “Average” cell’s area at different distances over time. Same data as presented in Figure 4, shown with different visualization. It can be seen that most morphological alterations occurs for HGF/SF-treated cells far from the wound at the later stages of healing. (b) Estimated density as function of time for close (<248 µm, red markers) and far (>248 µm, green markers) cells. Throughout the healing process, untreated cells that are close to the wound’s edges are consistently spread sparsely compared to distance cells. During Phase 1 treated cells maintain similar location-dependent characteristics to those described for untreated cells. In Phase 2, treated cells "switch" - distant cells become sparsely distributed compared to front cells. (TIF) [file pone.0044671.s003.tif]

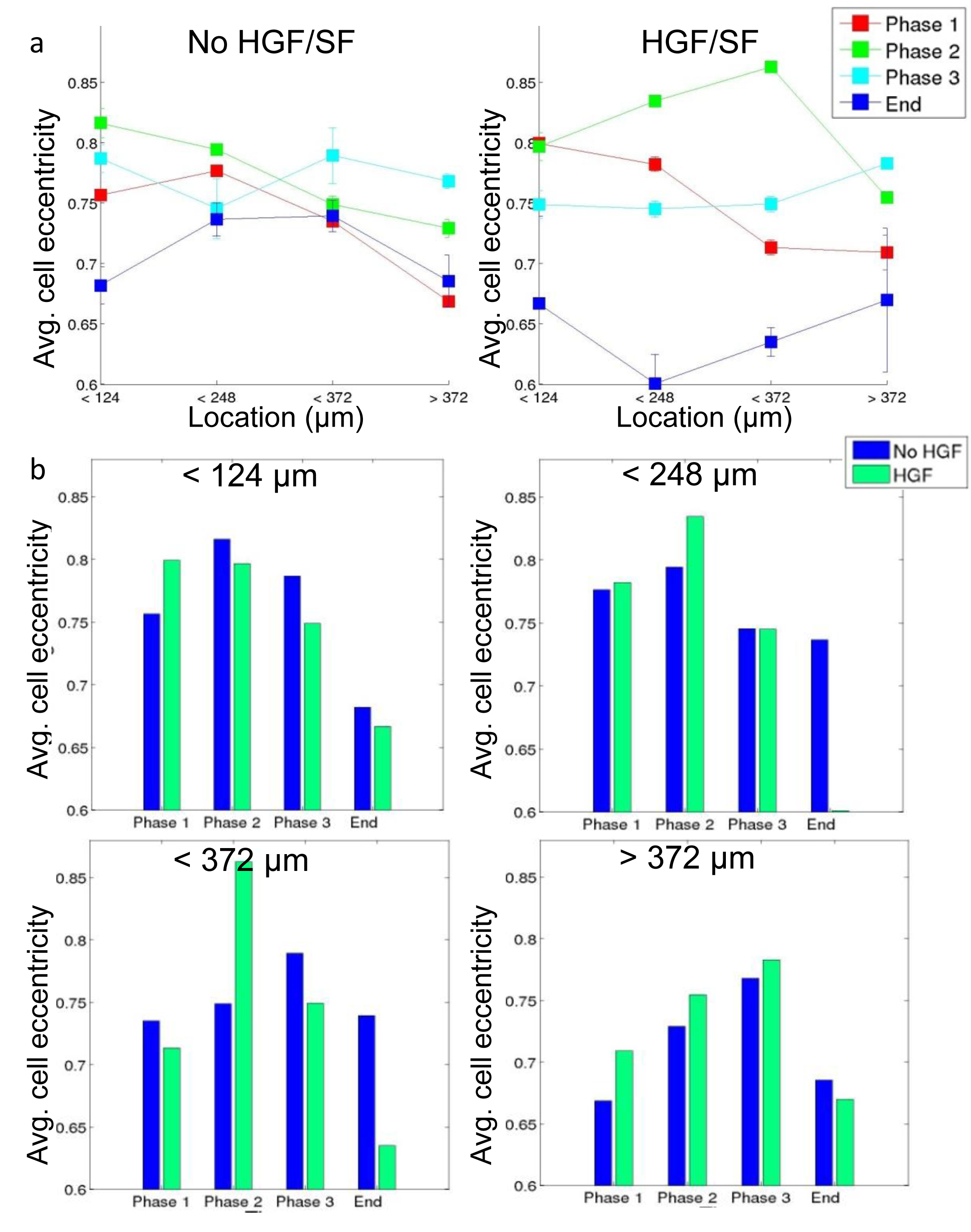

Supplement: Figure S4 — Single cell morphology (eccentricity) as function of time and distance from the wound. Eccentricity is the ratio of the distance between the foci of an ellipse and its major axis length. In our setting it is referred to the ellipse that has the same second-moments as the segmented cell. The values range between 0 and 1. (0 and 1 are degenerate cases; an ellipse whose eccentricity is 0 is actually a circle, while an ellipse whose eccentricity is 1 is a line segment.). (a) Same as Figure 6 a–b only for eccentricity instead of area: “Average” cell’s eccentricity at different distances over time. Untreated (left), and HGF/SF-treated cells (right). The x-coordinates represent discrete distance-intervals from the wound edge, the y-coordinates are the average cells’ eccentricity at a given distance interval and at a given phase in the healing process. Color markers represent the phase in the healing process. (b) Same as in Figure S3a only for eccentricity: “Average” cell’s eccentricity at different distances over time (different visualization). (TIF) [file pone.0044671.s004.tif]
