# Supplementary material for: Emergence of HGF/SF-Induced Coordinated Cellular Motility
Source: PLoS One. 2012 Sep 6;7(9):e44671. doi: 10.1371/journal.pone.0044671 (PMC3435317; doi:10.1371/journal.pone.0044671)
Supplement: Methods S1 — In this document we provide a detailed description of the following methods: (1) velocity magnitude map, (2) fully-automated single cell trajectory-estimation from DIC-based motion vector fields, (3) semi-automated single cell tracking, (4) velocity magnitude map based on semi-automated single cell tracking, and (5) single-cell morphology measures. (DOCX) [file pone.0044671.s005.docx]

Emergence of HGF/SF induced Coordinated Cellular Motility – Supporting Methods

Assaf Zaritsky^1a^, Sari Natan^2 a^, Eshel Ben-Jacob^3^, and Ilan Tsarfaty^2*^

^1^ Blavatnik School of Computer Science, Tel Aviv University, Tel Aviv, Israel,

^2^ Department of Clinical Microbiology and Immunology, Sackler School of Medicine, Tel Aviv University, Tel Aviv, Israel,

^3^ School of Physics and Astronomy, Tel Aviv University, Tel Aviv, Israel.

**Velocity Magnitude Map – Fully Automated Objective Multi-Cellular Motility Representation and Visualization of Wound Healing Time Lapse Experiments:**

*Segmentation of DIC image to cellular/background regions*: The *MultiCellSeg* segmentation algorithm [[1](#_ENREF_1)] is based on statistical learning of the local appearance of cellular versus background (non-cell) small image-regions (denoted *patches*) in wound healing assay DIC images. The algorithm is composed of three steps: (1) Classification of patches to "cellular" or "background" by a pre-trained linear patch classifier (2) Reclassification of regions (spatial grouping of neighboring patches) tagged as background, to discard cell regions classified incorrectly as background by the patch classifier using a pre-trained region classifier (3) Refinement of the extracted contours using graph-cut segmentation.

*Local-motion estimation*: A grid of local patches of size ~18.5µm x 18.5µm each (15 x 15 pixels) is overlaid on each image from the time-lapse sequence. For every patch, motion estimation is computed on the consecutive frame using the Bhattacharyya distance measure, proven beneficial for tracking tasks [[2](#_ENREF_2)]: the normalized patch is searched for in the corresponding time-lapse frame in a radius of ~12.5µm (10 pixels) from its original location, by maximizing the Bhattacharyya coefficient between the original patch and every possible shift in this radius, the inherent texture in DIC images allow to extract these measures directly. This step defines the motion-estimation vector for every patch in the time-lapse sequence. Velocity of each patch within cellular regions (defined by the image's segmentation) is computed from these motion estimations.

*Quantification of local-motion fields at a given distance from the wound*: MultiCellSeg is applied to define the current wound outlines. These contours were used to define a measure to estimate the velocity magnitude of the "average cell" located at a given distance from the wound; a *strip* at distance *d* is defined by a mask containing all pixels at a distance *d* from the wound edge. The average velocity magnitude of all pixels in a given strip estimates the average motility (speed) of cells at the corresponding distance from the wound edge. An illustration of the velocity magnitude estimation as function of distance from the wound is presented in Fig. 2.

*The "velocity magnitude map", cells velocity as function of time and distance from the wound:* A compact representation of a full-length time lapse experiment is defined by the *velocity magnitude map*, a two-dimensional depiction of the average motility of all cells at a given distance from the wound edge at a given time point. Extraction of the mean motility is accomplished for every time point and for a discrete quantization of distances from the wound to produce the velocity magnitude map. Thus, the *x*-axis represents time; the *y*-axis represents the distance from the wound. Each bin (*t*,*d*) in this map represents the average velocity magnitude (µm/hour) of cells at distance *d* from the wound at time *t*. The continuous representation of all time points in discrete distance quantization defines the complete map. Fig. 3a illustrates two representative maps of experiments untreated and treated with HGF/SF, the two vertical lines in each map define the partition to the three phases in the healing.

**Fully-Automated Single Cell Trajectory-Estimation from DIC-based Motion Vector Fields:** Five iterations of bilateral filtering [[3](#_ENREF_3)] were applied on the local motion estimation grid for each motion estimation frame from the time-lapse experiment. Given an initial cell location at position (x,y) at time t, its estimated trajectory is defined by iteratively placing (x,y) to its estimated location in the next frame by applying the filtered motion estimation.

**Semi-Automated Single Cell Tracking:** Three distances intervals from the initial wound were defined at 0-100µm, 100-200µm, and farther than 200µm. In each distance interval, several (5-21) cells were selected and tracked throughout the healing process. The tracks were built using the tracking & analysis software, ImarisTrack module, Imaris version 7.1.1. Cells trajectories were reviewed by an expert eye determining that each cell is tracked correctly using visual information both from the DIC and fluorescent images. Algorithmic tracking errors were corrected manually by severing the mistaken trajectory and manually tagging the next few correct locations before enabling the Imaris algorithm to continue tracking. The velocity component of each cell toward the wound was recorded based on a sliding window of 5 time frames (to cancel algorithmic tracking noise). The average velocity component toward the wound (Y-axes in our experiments) of cells at a given distance interval is presented in Fig. 3c, this component was selected to emphasis the phenomenon signal.

**Velocity Magnitude Map based on Semi-Automated Single Cell Tracking:** An alternative velocity magnitude map is constructed from single cell trajectories that were tracked ( Imaris) semi-automatically. An empty map is initiated, and each tracked cells contributes to the relevant bins based on its local speed and distance from the wound (defined by the MultiCellSeg's segmentation). Two examples of single cell based velocity magnitude maps are illustrated in Fig. 3b.

**Single-Cell Morphology Measures**: Analysis of cellular morphology was performed by statistical measurement of characteristics at the single cell level. A manual marking tool was developed to allow fully blinded manual segmentation of single cells in wound healing DIC images. An expert biologist manually segmented the exact contours of about 40 cells per image under different treatments, at different phases in the healing and at arbitrary locations. By introducing partial field-of-view, the biologist was not aware of the marked cell's origin (treatment, location, time). Two measures were considered: the size (area) of cells and their eccentricity (measure for roundness: low values – more rounded, high value – more elliptical). These measures were later used to extract quantitative statistical description of cells morphology under varying conditions in time, location and treatment.

Cells density was estimated directly from the cells' area. At a given time and distance interval, density was defined as the number of cells that fit in a given area unit based on the average cells' size. "Close cells" were defined as cells up to 248µm from the wound, while "distance cells" where defined to be at least 248µm away from the wound's edge.

**References**

1. Zaritsky A, Natan S, Horev J, Hecht I, Wolf L, et al. (2011) Cell motility dynamics: a novel segmentation algorithm to quantify multi-cellular bright field microscopy images. PLoS One 6: e27593.

2. Comaniciu D, Ramesh V, Meer P. Real-Time Tracking of Non-Rigid Objects using Mean Shift; 2000; Hilton Head Island, South Carolina. pp. 142-149.

3. Tomasi C, Manduchi R. Bilateral Filtering for Gray and Color Images; 1998; Bombay, India.
